# Functional connectivity of fMRI using differential covariance predicts structural connectivity and behavioral reaction times

**DOI:** 10.1162/netn_a_00239

**Published:** 2022-06-01

**Authors:** Yusi Chen, Qasim Bukhari, Tiger W. Lin, Terrence J. Sejnowski

**Affiliations:** Computational Neurobiology Laboratory, Salk Institute for Biological Sciences, La Jolla, CA, USA; Division of Biological Studies, University of California San Diego, La Jolla, CA, USA; McGovern Institute for Brain Research, Massachusetts Institute of Technology, Cambridge, MA, USA; Neurosciences Graduate Program, University of California San Diego, La Jolla, CA, USA; Institute for Neural Computation, University of California San Diego, La Jolla, CA, USA

**Keywords:** Functional connectivity, Resting-state fMRI, Differential covariance, Human Connectome Project

## Abstract

Recordings from resting-state functional magnetic resonance imaging (rs-fMRI) reflect the influence of pathways between brain areas. A wide range of methods have been proposed to measure this functional connectivity (FC), but the lack of “ground truth” has made it difficult to systematically validate them. Most measures of FC produce connectivity estimates that are symmetrical between brain areas. Differential covariance (dCov) is an algorithm for analyzing FC with directed graph edges. When we applied dCov to rs-fMRI recordings from the human connectome project (HCP) and anesthetized mice, dCov-FC accurately identified strong cortical connections from diffusion magnetic resonance imaging (dMRI) in individual humans and viral tract tracing in mice. In addition, those HCP subjects whose dCov-FCs were more integrated, as assessed by a graph-theoretic measure, tended to have shorter reaction times in several behavioral tests. Thus, dCov-FC was able to identify anatomically verified connectivity that yielded measures of brain integration significantly correlated with behavior.

## Author Summary

Our ability to sense, think, feel and react is reflected in the activation patterns of distinct brain regions. These patterns can be observed noninvasively by functional magnetic resonance imaging (fMRI). Statistical dependencies between the activities in brain regions have been widely used to measure functional connectivity (FC). However, due to common inputs from other brain areas, two correlated brain regions may not have direct neural fibers connecting them, which makes correlated activity difficult to interpret. A new method for measuring FC called differential covariance (dCov) was applied to fMRI recordings from anesthetized mice and resting human subjects. The analysis closely matched known neuronal fiber connections. In addition, dCov-FC from individual humans predicted reaction times for several types of psychological tests.

## INTRODUCTION

Functional connectivity (FC) is a measure of how brain regions interact with each other during resting-state functional magnetic resonance imaging recordings (rs-fMRI). The definition of FC has shifted from association to causation, which was previously referred to as effective connectivity (Reid et al., 2019). To be consistent with the prevailing definition, we refer to all estimated connectivity patterns, whether causal or not, as FC. Methods for computing FC have been tested on synthetic data, but testing on fMRI recordings is problematic without ground truth. New approaches are needed using other sources of evidence.

Promising new methods have been developed for extracting FC from fMRI data, including Granger causality (Granger, 1969) and dynamic causal modeling (DCM) (Friston, Harrison, & Penny, 2003). Granger causality uses a linear autoregressive model to quantify connectivity in terms of temporal dependencies. DCM uses a generative model to infer the causal latent states underlying the observations. Regression dynamic causal modeling (rDCM) was recently developed assuming that the dynamics are linear. These methods have advanced our understanding of FC but they are computationally demanding and have a large number of parameters (K. Stephan, Iglesias, Heinzle, & Diaconescu, 2015). Here we explore a new FC method that is scalable to very large datasets while maintaining good performance.

The covariance matrix is the most commonly used method to calculate FC and is computationally efficient (Smith et al., 2011); however, two correlated nodes may not have a direct physical connection because of covariance propagation (Stevenson, Rebesco, Miller, & Körding, 2008). Partial covariance reduces indirect correlations but not interactions due to unobserved common inputs. These spurious connections produced by covariance-based methods make it harder to interpret the links between FC and the underlying physiological states. In addition, covariance is a symmetric matrix, which is not consistent with functional dependencies that are not symmetric.

**Figure 1.**
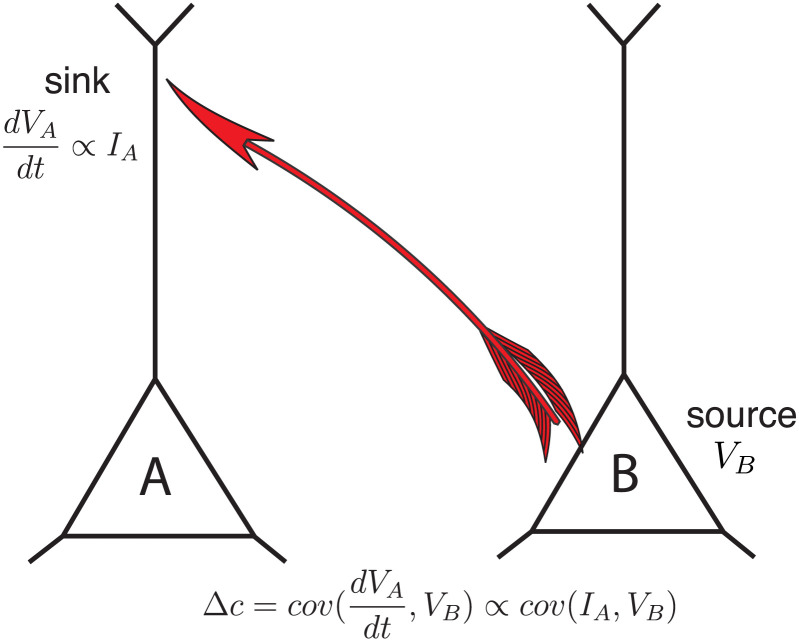
Differential covariance estimates the influence of source activity on changes in the sink activity. The output from the source area is driven by *V*_*B*_ and the input to the sink area is given by $\frac{{dV}_{A}}{dt}$, which is related to the input currents, *I*_*A*_, by the Hodgkin–Huxley equation. The covariance between these two signals is a measure of the directed flow of information from the sources to the sinks.

Differential covariance (dCov), which is the focus of this study, reduces false positive connections and recovers the ground truth connectivity from data generated by simulated models (Lin et al., 2020; Lin, Das, Krishnan, Bazhenov, & Sejnowski, 2017).^1^ Compared with other statistical methods, dCov seeks to estimate connectivity from a dynamical system perspective. It makes use of the derivative signal, in addition to the original signal, to capture the dynamical interactions between neural measurements and is, in general, not a symmetric matrix. When recorded neural responses are viewed as a dynamical system governed by a set of ordinary differential equations, the derivative provides information about the moving trend of the state variables. By evaluating the relationship between the derivative signal and the original signal, we can link the current state to future states, and thus extract the directed connectivity pattern (Figure 1). The goal of this article is to apply dCov to rs-fMRI recordings to evaluate its performance compared with other FC methods in predicting structural connectivity and behavioral data.

Some studies have focused on validating FC on several known projections or subnetworks, such as the default mode network (DMN) (Park, Friston, Pae, Park, & Razi, 2018), but leave open the validity of the larger FC matrix. Since direct activity dependencies between two brain regions depend on fiber projections connecting them, it is reasonable to expect the FC to be closely related to the underlying structural connectivity (SC) (Vincent et al., 2007), with the strength depending on brain dynamics. We used SC obtained through either viral tracing in mice or diffusion magnetic resonance imaging (dMRI) in humans as surrogates for “ground truth.”

Although the existence of anatomical connections, either direct or indirect, is a necessary condition for the dynamic coupling of neuronal activities between two brain regions, neural activity dependencies are more dynamic and variable than the underlying anatomical linkages. Honey et al. (2009) systematically examined the relationship between Pearson correlation–based FC and diffusion tensor imaging (DTI) based SC at high spatial resolution. They pointed to the redundancy and unreliability of structurally unconnected functional connections. Since then, major efforts have been made to predict FC from SC (Abdelnour, Dayan, Devinsky, Thesen, & Raj, 2018; Abdelnour, Voss, & Raj, 2014; Grandjean, Zerbi, Balsters, Wenderoth, & Rudin, 2017; Messé, Rudrauf, Giron, & Marrelec, 2015). But these approaches typically accounted for less than half of the known structural connections (Grandjean et al., 2017). A complete match from the entire weighted FC matrix to the weighted SC matrix is difficult to achieve owing to limitations of both FC estimation and DTI. To obtain a better match between FC and SC, we chose to focus on examining the subset of significant functional connections. This also had the advantage of increasing the tolerance of FC estimation to various noise sources in fMRI recordings (Liu, 2016).

Another challenge is to gain insights from the estimated FCs into how behavior is generated in brains. Methods from network science, especially those rooted in graph theory, have been useful for analyzing the dynamics of how information is communicated in brains (Avena-Koenigsberger, Misic, & Sporns, 2018). Several core concepts describing the segregation and integration of information (Sporns, 2013) have provided insights into cognitive control mechanisms (Cohen & D’Esposito, 2016; Marek, Hwang, Foran, Hallquist, & Luna, 2015; Santarnecchi, Galli, Polizzotto, Rossi, & Rossi, 2014) and brain disorders (Huang, Wu, Lin, Pang, & Chen, 2019). This suggests that the topological properties of FCs evaluated by different methods could provide new insights into brain function.

To extract the most reliable signals, we binarized FC and SC and systematically compared FC defined by dCov with commonly used FC estimation methods, quantifying performance by their correspondence with anatomically strong connections. The resulting FC matrices were also topologically analyzed and compared with behavioral measurements to link them with brain states that give rise to behavior.

## COMPUTATIONAL BACKGROUND

The differential covariance method makes use of the derivative signal. In most models of neural dynamics, the derivative of a neuron’s membrane potential is related to its incoming synaptic and intrinsic currents (i.e., sinks), and its own membrane potential is related to its output (i.e., sources) (Figure 1). Differential covariance uncovers the relationship between sinks and sources. In this paper, we calculated three dCov matrices: differential covariance matrix, Δ*c* (Supporting Information Figure S1, Equation 1); partial differential covariance matrix, Δ*p* (Supporting Information Figure S1, Equation 2); and sparse-latent regularized partial differential covariance matrix, Δ*s* (Supporting Information Figure S1, Equation 3). We briefly introduce here these three measures and further discuss them along with other measures in the Methods section below.

The differential covariance Δ*c* is defined in Equation 1, where *z*_*i*_ is the time trace of node *i* in a network, *dz*_*i*_ is the numerical derivative of *z*_*i*_ (the superscript bar denotes the sample mean of a time trace), and *cov* denotes the estimation of sample covariance between two time traces.

$$\Delta c_{ij}=cov\left({dz}_{i},z_{j}\right)=\frac{1}{T-1}\sum_{t=1}^{T}\left({dz}_{i}\left(t\right)-{\bar{dz}}_{i}\right)\left(z_{j}\left(t\right)-{\bar{z}}_{j}\right)$$ (1)

The partial differential covariance Δ*p* can be calculated in Equation 2, where *K* is the set of nodes other than *i* and *j*; Cov is the covariance matrix; Cov_*jK*_ ∈ ℝ^1×(*N*−2)^, Cov_*KK*_ ∈ ℝ^(*N*−2)×(*N*−2)^; and Δ_*ciK*_ ∈ ℝ^1×(*N*−2)^.

$$\Delta p_{ij}=\Delta c_{ij}-{Cov}_{jK}{Cov}_{KK}^{-1}\Delta c_{iK}^{T}$$ (2)

The partial differential covariance controls the effects of confounding variables by calculating the relationship based on residual time traces, paralleling the derivation of the partial covariance matrix (Cox & Wermuth, 2014). In most cases, the residual time traces were obtained by solving a multiple linear regression problem with confounding variables included as independent variables.

The third matrix, Δ*s*, minimizes the effects of latent inputs from outside the recorded regions, which are not uncommon in fMRI recordings. We adopted a sparse latent regularization method to separate Δ*p* into a sparse matrix Δ*s* and a low-rank matrix *L*. Under reasonable assumptions, the sparse matrix represents direct coupling between observed nodes and the low-rank matrix represents the residual effects from unobserved nodes (Yatsenko et al., 2015). We obtained Δ*s* by solving a constrained optimization problem using an augmented Lagrangian multiplier (Candès, Li, Ma, & Wright, 2011). The objective function is the weighted sum between L1-norm of the vectorized sparse matrix and the trace of the low-rank matrix. The penalty ratio *α* controls the sparsity of Δ*s*. We fixed *α* to $\frac{1}{\sqrt{N}}$ because this choice provides an almost exact solution to the sparse matrix separation problem (Theorem 1.1 in Candès et al., 2011):

$$\begin{array}{l} \text{Constrain}:\Delta p=\Delta s+L\\ \Delta s=\arg\;\min_{\Delta s}\left({\left\|\Delta s\right\|}_{1}+\alpha *tr\left(L\right)\right) \end{array}$$ (3)

**Figure 2.**
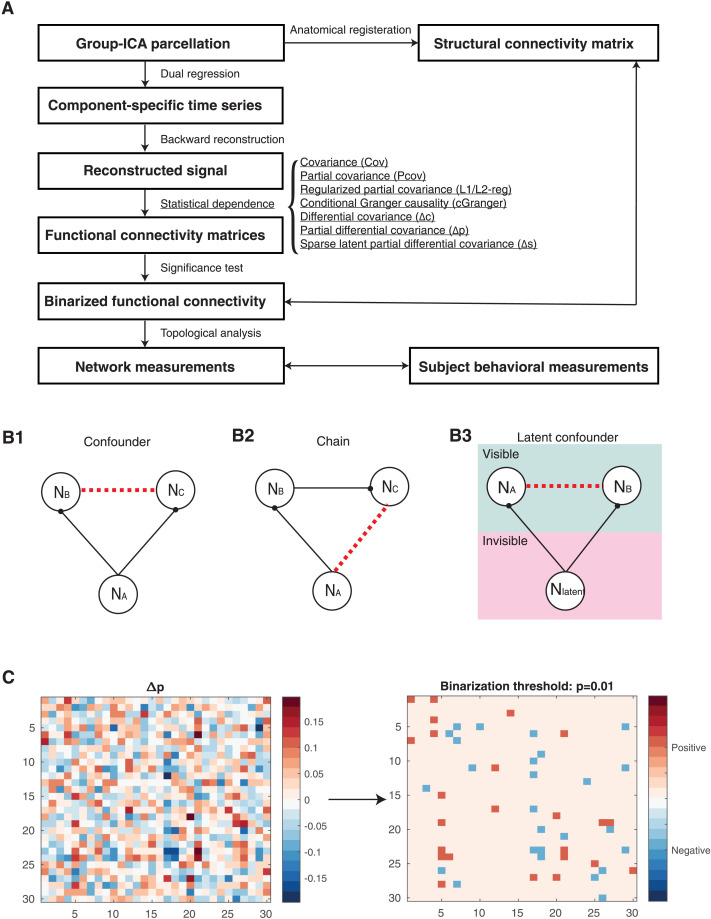
Workflow and features of differential covariance-based methods. (A) Workflow. Two-way arrows mean that we compared the boxed quantities. (B) Differential covariance-based methods have been shown to effectively reduce the three types of errors in network modeling shown in B1, B2, and B3 (Lin et al., 2017). Black solid lines denote actual physical connections and red dashed lines denote false positive correlational relationship. B1: confounder effects due to common input; B2: chain effects due to propagation; B3: latent confounders due to unobserved common inputs. (C) The partial differential covariance matrix, Δ*p*, on the left and its binarized matrix on the right (binarization threshold = 0.01) of one mouse subject. Partial differential covariance matrices are directed, nonsymmetric, and sparser than the corresponding covariance matrices (Supporting Information Figure S1).

These three differential covariance matrices reduce three types of common false positive connections (Figure 2B). To be more specific, Δ*c* deals with errors due to confounder effects (Figure 2B1). In the calculation of Δ*p*, both confounder (Figure 2B1) and chain effects (Figure 2B2) are taken into account. The sparser Δ*s* further reduces the effect of latent confounders (Figure 2B3).

## RESULTS

### Functional Connectivity Estimated by Differential Covariance

**Figure 3.**
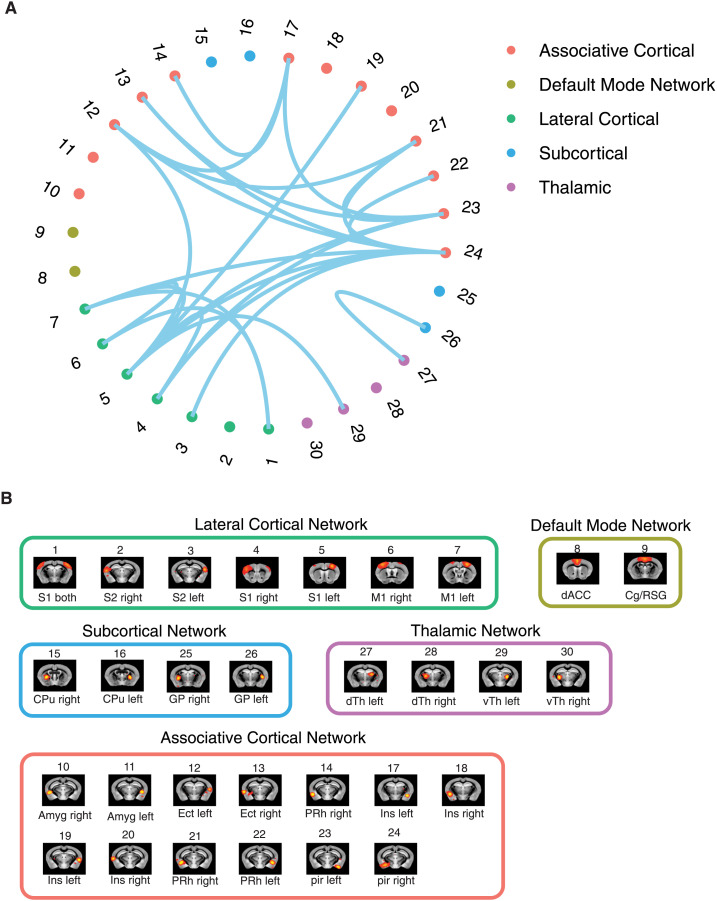
Significant Δ*p*-FC connections shared across subjects and associated MRI spatial maps from the mouse rs-fMRI dataset. (A) For Δ*p*-FC estimated from the mouse dataset, connections which are significant (binarization threshold = 0.01) in more than 2 subjects (out of 12) were plot in the circular plot. (B) MRI spatial maps of the independent components were grouped into subnetworks. The registered anatomical locations were shown underneath. Note that there could be more than one component (for example, component 17 and 19) corresponding to the same anatomical region due to the unsupervised nature of group ICA. S1, primary somatosensory cortex; S2, secondary somatosensory cortex; M1, primary motor cortex; dACC, dorsal anterior cingulate cortex; Cg, cingulate cortex; RSG, retrosplenial cortex; Amy, amygdalar; Ect, entorhinal cortex; PRh, perirhinal; CPu, caudoputamen; Ins, insular; pir, piriform; GP, Globus Pallidus; dTh, dorsal thalamus; vTh, ventral thalamus. Refer to Supporting Information Table S3 for detailed annotations.

**Figure 4.**
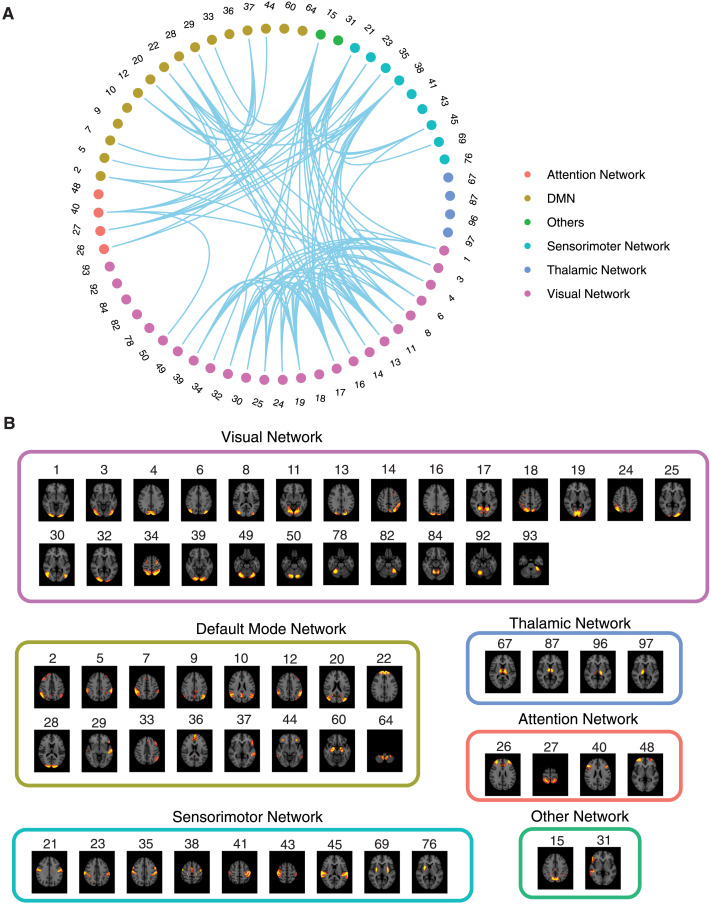
Significant Δ*p*-FC connections shared across subjects and associated MRI spatial maps from the HCP rs-fMRI dataset. (A) For Δ*p*-FC estimated from the HCP dataset, connections that are significant (binarization threshold = 0.01) in more than 30 subjects (out of 1,003) were plot in the circular plot. (B) MRI spatial maps of the independent components were grouped into subnetworks. The number marks the index of the independent components. The indices are from 1 to 100, but only 60 of them were shown here because the remaining components were treated as imaging artifacts. Refer to Supporting Information Table S2 for more detailed annotations.

We followed standard procedures for preprocessing fMRI recordings (Smith et al., 2013). Voxel-wise time traces were decomposed into component-wise time traces and MRI maps of each component through group independent component analysis (ICA) and dual regression (Figure 2A) (Methods). After manually removing artifactual components, the remaining components (30 for mouse and 60 for HCP) were registered with corresponding anatomical locations in a brain atlas (MRI maps are shown in Figure 3 and Figure 4; annotations are shown in Supporting Information Table S2 and Table S3). This preprocessing yielded an *N* × *T* (number of components × number of time points) matrix representing time-varying haemodynamic signals for various anatomical locations. We then applied a recently developed backward reconstruction method to reconstruct neural signals (*z*) from haemodynamic signals (example time traces shown in Supporting Information Figure S4). Backward reconstruction based on the forward Balloon model (Friston, Mechelli, Turner, & Price, 2000) has been shown to work well together with dCov applied to synthetic datasets (Lin et al., 2020).

Functional connectivity (FC) matrices (*N* × *N*) were estimated from the reconstructed neural signals, *z*, using popular FC estimation methods, together with the three dCov matrices mentioned above. The benchmarked estimators (Methods) include the sample covariance matrix (Cov), the precision matrix (Pcov), L1-/L2-regularized precision matrix (L1-/L2-reg), and conditional Granger causality (cGranger). In general, dCov matrices give rise to FC matrices that are not symmetric, and are antisymmetric for Δ*c*, which could, in practice, potentially predict the direction of information flow (Supporting Information Figure S1).

To reduce the susceptibility of FC matrices to noise, we determined the significance level of connections by using a bootstrap procedure (except for cGranger whose significance level was calculated through *χ*^2^ distribution). The null hypothesis is that activities of all the nodes in the network are generated independently and, as a consequence, there are no statistical relationships between any node pair within the null dataset. An autoregressive (AR) bootstrap procedure (Efron & Tibshirani, 1986; Nalci, Rao, & Liu, 2019) was used to generate the null time traces to preserve the power spectral density of haemodynamic signals, and then null FCs were estimated from the null time traces. For each empirical connection, the probability that its value belongs to the null distribution, its *p* value, was calculated assuming the distribution was Gaussian. Thresholds of significance were applied to binarize the FC matrix so that we could focus on a set of significant connections. Because dCov-based methods intrinsically reduce false positive connections, they yielded fewer significant connections compared to covariance-based methods (Supporting Information Figure S1).

We applied the above workflow to extract FCs from fMRI recordings in anesthetized mice and resting-state recordings from the HCP dataset. The mouse dataset included 12 subjects, and the HCP dataset included 1,003 young adult subjects. Figures 3B and 4B show the MRI spatial maps of each component. Due to the individual variability of connectivity patterns, the circular plots in Figures 3A and 4A only showed the subset of significant connections shared across subjects. Components were allocated into networks according to their corresponding anatomical locations. The first inspection of shared connections showed promising correspondence between Δ*p*-FC and the anatomical wiring of different brain regions. For example, in Figure 3, Δ*p*-FC picked up important connections within the mouse lateral cortical network, which is located in the densely interconnected Module 2 (M2) subsystem reported by Swanson et al. (2018) (M2 is shown in Figure 3 of the reference paper). In Figure 4, the visual network is densely interconnected across multiple subjects revealed by Δ*p*-FC. Therefore, we next quantified the similarity between dCov-FC and SC at individual level and population level in human and mouse subjects, respectively.

### Differential Covariance FC More Closely Matches the Underlying Structural Connections

We developed a pipeline to systematically quantify the similarity between FC and SC. We first constructed an *N* × *N* structural connectivity, “ground truth” matrix, with nodes corresponding to the anatomical regions that matched the independent components obtained from resting-state recordings (Supporting Information Figure S2 and Figure S3, Methods). We used the existing SC database for the connectivity profiles between different anatomical regions. Specifically, we used population level viral tracing data (Swanson et al., 2018) for mouse SC and individual level dMRI data (Rosen & Halgren, 2021) for human SC (Methods). The mouse “ground truth” matrix (Supporting Information Figure S2) is not symmetric while the human “ground truth” matrices (Supporting Information Figure S3) are symmetric and sparse with substantial amount of individual variability. In both cases, higher values indicate higher structural connectivity strength. We then calculated the average structural connectivity strength (ASCS) of the set of significant connections picked through AR bootstrapping under multiple binarization thresholds. The decreasing binarization threshold provides a stricter criterion for connection selection and pictures an asymptotic description of the evaluated quantity. A higher ASCS value implies that a specific FC more closely matched the underlying SC.

**Figure 5.**
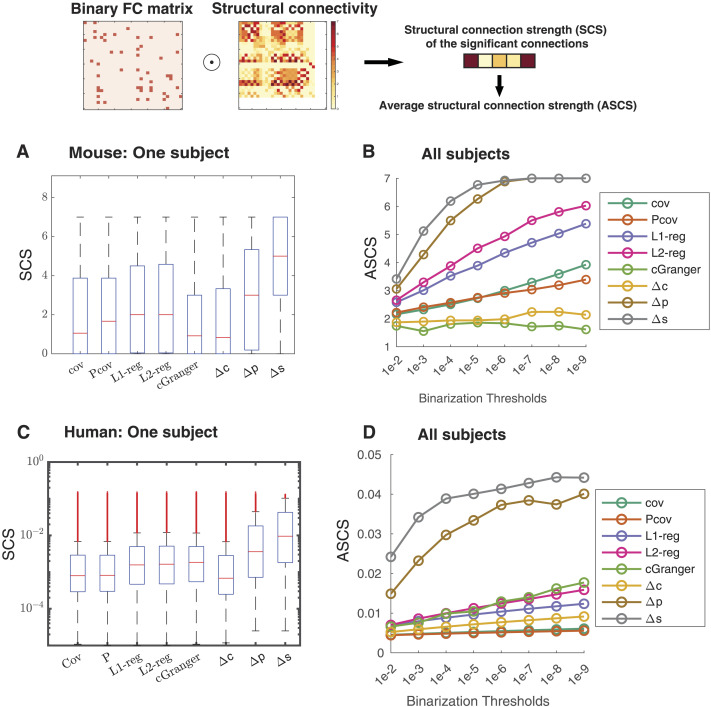
Differential covariance (dCov)-FC preferentially identified connections with high SCS in both mouse and HCP dataset. (A and C) The SCS distribution of the significant (binarization threshold: *p* < 0.01) connections identified by different methods from recordings of one mouse subject (A) or one human subject in the HCP dataset (C). Δ*p* and Δ*s* identified connections with higher SCS. (B and D) Under different binarization thresholds, ASCS of the significant connections pooled from all subjects in the mouse dataset (B) and the HCP dataset (D). Under all binarization thresholds, Δ*p* and Δ*s* have better performance than the baseline methods in picking up structurally connected regions. FC, functional connectivity; cov, covariance; Pcov, partial covariance; L1/L2-reg, L1/L2-norm regularized partial covariance; cGranger, conditional Granger causality; Δ*c*, differential covariance; Δ*p*, partial differential covariance; Δ*s*, partial differential covariance with sparse latent regularization.

We calculated ASCS values for FC defined by the covariance matrix (cov), partial covariance matrix (Pcov), regularized partial covariance matrix (L1/L2-reg), conditional Granger causality (cGranger), differential covariance matrix (Δ*c*), partial differential covariance matrix (Δ*p*), and sparse differential covariance matrix (Δ*s*). Figure 5B and 5D show ASCS values of significant connections, using multiple binarization thresholds, pooled from all subjects in mouse and HCP dataset, respectively. In both mouse (Figure 5B) and human datasets (Figure 5D), Δ*p* and Δ*s* had significantly (*p* < 0.05, rank-sum test) higher ASCS values than those based on benchmark methods. Figure 5A and 5C showing structural connectivity strength (SCS) distributions from one individual subject revealed a reduced number of low-SCS connections in both Δ*p* and Δ*s*. This is a consequence of the method’s ability to reduce false positive connections, as previously shown using synthetic data in simulation studies (Lin et al., 2020). In general, dCov-FC produced sparse estimation, which contributed to its higher ACSC values. Even at the same sparsity level, Δ*s* still showed significantly higher ASCS compared to all other methods (Supporting Information Figure S7). This means that in addition to sparsity, there are other favorable statistical properties that enable dCov to capture important anatomical connections.

### Differential Covariance FC Measures the Integration of Brain Activity

Next, we wanted to know whether dCov-based FC matrices, which are aligned with the underlying SC, could also provide insights into the interpretation of behavioral measurements. The HCP dataset (Methods) consists of not only high-quality macroscopic-level imaging data, but also hundreds of behavioral measurements, including detailed psychological test results from 1,003 young adult subjects (Van Essen et al., 2013). The large sample size and the full assessment of subjects’ psychological states render the HCP dataset an invaluable source for investigating the organizing principles linking human brain networks and behavioral readouts.

**Figure 6.**
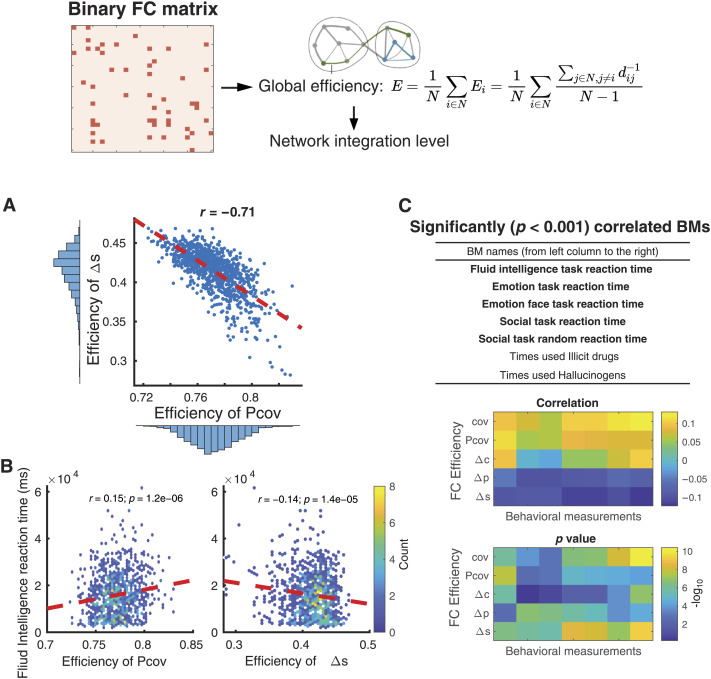
Differential covariance based FC matrices could be a more reasonable measurement of the integration level of the brain. (A) In the HCP dataset, global efficiency of Δ*s*-FC and Pcov-FC are strongly anti-correlated (*r* = 0.71). (B) Global efficiency of Pcov-FC is positively correlated (left, *r* = 0.15, *p* = 1.2e−6) to the reaction time of fluid intelligence task while the global efficiency of Δ*s*-FC is negatively correlated (right, *r* = −0.14, *p* = 1.4e−5) to it. In most cases, we would expect a negative correlation because the more highly integrated the network, the shorter the response time. Color scale codes for the density of the samples. (C) The table lists the behavioral measurements that are most significantly correlated (*p* < 0.001) to the global efficiency of Δ*s*-FC, after controlling for confounding factors. Most of the significant behavioral measurements are related to reaction time in different tasks (shown in bold). Heat maps below show their correlation values and *p* values.

We calculated several topological properties of the FC matrices (Rubinov & Sporns, 2010). Because of the inherent interdependencies and transitivity of bivariate methods, the covariance matrix is not suitable for calculating network properties (Avena-Koenigsberger et al., 2018). So FC derived from the partial correlation matrix (Pcov-FC) was used for control. Pcov-FC and Δ*s*-FC exhibited similar network properties such as network degree distribution (Supporting Information Figure S5A), clustering behavior, transitivity, and modularity properties (Supporting Information Figure S5C) across subjects. In addition, both Pcov-FC and Δ*s*-FC showed similar network degree distribution to that of population level diffusion tensor imaging network reported in Hagmann et al. (2008) (Supporting Information Figure S5B). Surprisingly, the network efficiency of Δ*s*-FC is strongly anticorrelated (*r* = 0.71) with that of Pcov-FC (Figure 6A). Network efficiency, calculated as the average of the inverse path length of shortest signaling routes between two nodes (Methods), measures the network’s integration level. Since Δ*s*-FC reduces false positive connections/paths in the network, it selects for the important and reliable routes and decreases redundant routes. As a consequence, network efficiency of Δ*s*-FC could potentially be a better description of the integration level.

To investigate this hypothesis, we compared this measure of network integration with each subject’s fluid intelligence quantified by their reaction time to the test. This behavioral measurement has been previously shown to be most related to FC (Smith et al., 2015). One expects that a higher level of network integration would lead to shorter reaction times, and indeed Δ*s*-FC–based global efficiency is inversely correlated (*r* = −0.14) to reaction time (Figure 6B). As a control, we sorted the correlations between Δ*s*-FC–based global efficiency and all behavioral measurements after controlling common influencing factors like age and brain size. Five out of seven significant (*p* < 0.005) behavioral measurements are reaction time in various psychological tests (Figure 6C), which further confirmed the significance of Δ*s*-FC as a measure of the integration of brain activity.

## DISCUSSION

In this article, we applied three variants of dCov to estimate FC from rs-fMRI recordings. Compared to the covariance matrix, differential covariance identified a subset of connections with high structural connectivity strength, largely owing to dCov’s ability to reduce three types of false positive connections. These results were confirmed in both human and mouse recordings. In addition, by ruling out redundant signaling routes, the sparse-latent regularized partial differential covariance matrix (Δ*s*-FC) provided the closest link to behavioral measurements and functional network integration based on the inverse of shortest path length, a measure of global efficiency.

The estimates of directed connectivity afforded by dCov-FC are formally related to the estimates of effective connectivity from dynamic causal modeling, under linear assumptions, because dCov-FC rests upon the same generative model used in rDCM (Frässle et al., 2017). However, interactions are estimated using variational Bayesian inference in dynamic causal modeling (Friston et al., 2003), whereas dCov-FC expresses the interaction pattern in terms of the covariance between the derivative signal and the original signal. Without the need to go through the model fitting process, dCov is a much faster and more straightforward algorithm.

The current versions of dCov assume that the underlying system is linear and that the connectivity pattern is closely related to the adjacency matrix in graph theory or transition matrix in dynamical system analysis. Although the computational processes in brains are by no means linear, the averaging of activity in large neural populations, as in the case of fMRI recordings, is approximately linear (Stephan et al., 2008). The current linear dCov estimator could be extended to nonlinear estimators, taking into account the threshold firing properties of individual neurons, to better extract connectivity at the single neuron level (Chen, Rosen, & Sejnowski, 2021).

The calculation of sparse-latent regularized partial differential covariance matrix (Δ*s*) used a method for sparse-latent regularization that was initially proposed by Yatsenko et al. (2015) and Candès et al. (2011). This sparse-latent regularization assumes a sparse structure of the intrinsic connections and a low-rank component representing common fluctuations and common latent inputs. This assumption may or may not be fulfilled in fMRI recordings, and there is no straightforward way to test this assumption. Nonetheless, the addition of sparse latent regularization did not influence the qualitative conclusions (compare Δ*p* and Δ*s* in Figure 5 and Figure 6C).

There are potential biases that may be introduced by using structural connectivity as the ground truth. First, the connection strengths revealed by the tractography-based measurements used in the HCP dataset decay exponentially with tract length (Rosen & Halgren, 2021), thus likely underestimating long-distance connections (Jones, Knösche, & Turner, 2013); Second, we used individual-level dMRI matrices for the HCP dataset to account to individual variability. These matrices are sparser compared to the population average. Finally, dMRI fails to reveal the direction of structural connections and the population level SC matrix constructed from mouse viral tracing is almost symmetric. These concerns limited the accuracy of comparisons between the directed connections inferred by dCov methods and the dMRI dataset.

Recent efforts have been made toward a dynamic description of directed connectivity, such as dynamic effective connectivity (Zarghami & Friston, 2020), and dCov is another step toward this direction. First, the derivative signal reflects a dynamical description of the system (Chen et al., 2021) and is also used in regression dynamic causal modeling (rDCM) (Frässle et al., 2017). In comparison, dCov is more intuitive and faster than rDCM. Second, a sliding-window approach has been used to compute time-dependent covariance matrices (Hutchison et al., 2013), aiming to capture context-dependent fluctuations in the functional connectivity. However, due to the stationarity assumption required by sample covariance estimation, fluctuations in the covariance matrix may imply a violation of stationarity rather than a measure of switching between brain states. Nor does a static covariance matrix imply the absence of switching (Liegeois, Laumann, Snyder, Zhou, & Yeo, 2017). In contrast, dCov does not require stationarity so that, in principle, applying a sliding-window analysis together with dCov could provide a more accurate description of the changes in regional coupling.

In conclusion, in addition to validating dCov’s performance on fMRI recordings, we have also extended previous studies linking FC to connectivity and behavior. The analytic techniques for studying links to anatomy and behavior introduced here can be applied to other methods for determining FC and other datasets, which could provide stronger confidence in their interpretation.

## METHODS

### Data Acquisition and Preprocessing

We used two rs-fMRI datasets, including an anesthetized mouse dataset first reported in Bukhari et al. (2018) and the extensively processed young adult rs-fMRI S1200 dataset from Human Connectome Project (https://www.humanconnectome.org).

For the mouse dataset, experiment and imaging protocols were defined in the original publication. For all 12 animals, each subject received approximately 1.2% Isoflurane (Abbott, Cham, Switzerland) with a tolerance of 0.1% in a 20% oxygen/80% air mixture. The data was collected in two sessions, where the animals remained inside the scanner during the whole time. The total time series acquired during the two sessions were of 680-s length. Subjects were scanned using a Bruker Biospec 94/30 small animal MR system (Bruker BioSpin MRI, Ettlingen, Germany) operating at 400 MHz (9.4 T). A gradient-echo echo-planar imaging (GE-EPI) sequence has been used for rs-fMRI data acquisition with field of view = 16 × 7 mm^2^, matrix dimensions = 80 × 35, TR = 1 second, TE = 12 ms, flip angle = 60 degree. Each subject had 680 imaging frames collected.

All the preprocessing was performed using tools from FMRIB’s Software Library (FSL version 5). FSL’s recommended pre-processing pipeline was used. Motion correction, removal of nonbrain structures, high-pass temporal filtering with sigma = 75.0 s, prewhitening, and global spatial smoothing using a 0.2-mm Gaussian kernel was applied to increase signal-to-noise ratio as part of the preprocessing. After the preprocessing, the functional scans were aligned to the high-resolution anatomical AMBMC template (https://www.imaging.org.au/AMBMC/AMBMC) using linear affine and nonlinear diffeomorphic transformation registration as implemented in ANTs (ANTs. v 1.9; https://picsl.upenn.edu/ANTS/). We used FSL’s MELODIC for probabilistic independent component analysis. The multisession temporal ICA concatenated approach, as recommended for rs-fMRI data analysis, allowed to input all subjects from all the groups in a temporally concatenated fashion for the ICA analysis. ConcatICA yielded different activations and artifact components without the need of specifying any explicit time series model. A total of 100 independent components (IC maps) were extracted, and the mixture model approach was applied on these estimated maps to perform inference analysis. An alternative hypothesis test based on fitting a Gaussian/gamma mixture model to the distribution of voxel intensities within spatial maps 53 and 54 was used to threshold the IC maps. A threshold of 0.5 (*p* < 0.5) was selected for the alternative hypothesis in order to assign equal ‘cost’ to false positives and false negatives. Out of the 100 independent components (IC maps), only 30 were selected, while the components that overlapped with vascular structures and ventricles were excluded from further analysis. Similarly, regions at the brain surface, which are prone to be affected by the motion artifacts due to, for example, breathing, were excluded.

For the HCP dataset, we used the extensively processed “HCP1200 Parcellation + Timeseries + Netmats (1003 Subjects)” dataset available through the website. Detailed preprocessing and study design could be easily accessed through the website. In this release, 1,003 healthy adult human subjects (ages 22–37 years, 534 females) were scanned on a 3-T Siemens connectome-Skyra scanner (customized to achieve 100 mT m^−1^ gradient strength). Each subject underwent 4 × 15 minutes recording sessions with temporal resolution of 0.73 second and spatial resolution of 2 mm isotropic. Subject-specific measures including behavioral, demographic, and psychometric measures are available from the HCP data dictionary.

For imaging data processing, each 15-minute run of each subject’s rs-fMRI data was preprocessed according to Smith et al. (2013); it was minimally preprocessed (Glasser et al., 2013), and had artefacts removed using ICA + FIX (Griffanti et al., 2014; Salimi-Khorshidi et al., 2014). Intersubject registration of cerebral cortex was carried out using areal feature–based alignment and the multimodal surface matching algorithm (MSMAll) (Glasser et al., 2016; Robinson et al., 2014). Each dataset was temporally demeaned and had variance normalization and then fed into the MIGP algorithm, whose output is the top 4,500 weighted spatial eigenvectors from a group-averaged PCA (a very close approximation to concatenating all subjects’ time series and then applying PCA) (Smith, Hyvärinen, Varoquaux, Miller, & Beckmann, 2014). The MIGP output was fed into group-ICA using FSL’s MELODIC tool, applying at several different dimensionalities (D = 25, 50, 100, 200, 300). In our analysis, we used a 100-dimensional decomposition because the number of parcellation nodes used in multiple studies was typically around 100 (Dadi et al., 2019).

### Component Specific Time Series

For a given “parcellation” (group-ICA map), the set of ICA spatial maps was mapped onto each subject’s rs-fMRI time series data to derive one representative time series per ICA component per subject. This process was fulfilled by the standard “dual-regression stage-1” approach, in which the full set of ICA maps was used as spatial regressors against the full data (Filippini et al., 2009). This results in an *N* × *T* (number of components × number of time points) matrix for each subject. Thus, we consider each ICA component as a network “node.” These time series can then be used to reveal statistical relationship in a network, as will be described in section Estimation of Functional Connectivity.

### Reconstruction of Neural Activity

Before estimating the functional connectivity, we adopted two methods to reconstruct neural signals *z* from haemodynamic time series *y*, which was obtained from the dual regression process described above.

#### Backward reconstruction.

Backward reconstruction was developed based on a biophysical model which transforms neural activities to BOLD signals via a set of differential equations (Stephan, Weiskopf, Drysdale, Robinson, & Friston, 2007). One important step in this model links blood flow to BOLD signal through a Balloon model assumption. Therefore, in this article, we will refer to this entire biophysical process as the forward Balloon model. The backward reconstruction process linearizes the equations and derives a surrogate neural signal *z* as a linear combination of haemodynamic signals *y* and its *i*^*th*^ order derivatives *y*^(*i*)^ (Equation 4) (Khalidov, Fadili, Lazeyras, Van De Ville, & Unser, 2011). Note that according to the linearized Balloon model, the reconstructed surrogate signal *z* is a linear combination of real neural signal *V* and its first order derivative $\dot{V}$,

$$\begin{array}{l} z=q_{1}\dot{V}+q_{0}V\\ \;=p_{4}y^{\left(4\right)}+p_{3}y^{\left(3\right)}+p_{2}y^{\left(2\right)}+p_{1}\dot{y}+p_{0}y \end{array}$$ (4)

where the coefficients *p*_*i*_ and *q*_*i*_ are functions of biophysical parameters: *τ*, *ϵ*, *α*, *κ*, *γ*, *ϑ*_0_, *ρ*, *η*, and *V*_0_ (Equation 5). Some parameter values vary with respect to magnetic field strength (B). Please refer to Supporting Information Table S1 for annotations of these biophysical parameters and the specific values used in this paper. Most values were taken from Stephan et al. (2007) unless cited. We also demonstrated that the backward reconstruction process is robust to a wide range of parameter values in simulation tests (data not shown).

$$\begin{array}{l} q_{1}=-\left(k_{1}+k_{2}\right)\tau \eta -\left(k_{3}-k_{2}\right)\eta V_{0}\\ q_{0}=-\frac{\left(k_{1}+k_{2}\right)\left(\tau -1+\alpha \right)\eta }{\alpha \tau }-\frac{\left(k_{3}-k_{2}\right)\eta }{\tau }V_{0}\\ k_{1}=4.3\vartheta_{0}\rho TE\\ k_{2}=\epsilon r_{0}\rho TE\\ k_{3}=1-\epsilon \\ p_{4}=\tau \\ p_{3}=1+\frac{1}{\alpha }+\tau \kappa \\ p_{2}=\frac{1}{\tau \alpha }+\tau \gamma +\left(1+\frac{1}{\alpha }\right)\kappa \\ p_{1}=\left(1+\frac{1}{\alpha }\right)\gamma +\frac{\kappa }{\tau \alpha }\\ p_{0}=\frac{\gamma }{\tau \alpha } \end{array}$$ (5)

#### Blind deconvolution.

Blind deconvolution is an alternative method to remove effects of hemodynamic response from rs-fMRI recordings (Wu et al., 2013). The basic idea is to consider the fluctuations of rs-fMRI as ‘spontaneous event-related,’ individuate point processes corresponding to signal fluctuations with a given signature, extract a region-specific hemodynamic response function and use it for deconvolution. Details and implementation could be found in the original article (Wu et al., 2013).

### Estimation of Functional Connectivity

Functional connectivity is represented by a *N* × *N* matrix, which describes the statistical relationship of *N* node time series. To quantify this relationship, we adopted commonly used covariance-based algorithms, Granger causality, and three novel differential covariance based algorithms.

#### Covariance based methods.

Denote the entire *N* node time series as a *N* × *T* matrix with each row vector as *z*_*i*_, then entry (*i*, *j*) of the covariance matrix Cov ∈ ℝ^*N*×*N*^ could be calculated as follows:

$${Cov}_{ij}=cov\left(z_{i},z_{j}\right)=\frac{1}{T-1}\sum_{k=1}^{T}\left(z_{ik}-{\bar{z}}_{i}\right)\left(z_{jk}-{\bar{z}}_{j}\right)$$ (6)

where the superscript bar denotes the sample mean of a time trace and *cov* denotes the estimation of sample covariance between two time traces.

The partial covariance matrix Pcov ∈ ℝ^*N*×*N*^, also called the precision matrix, aims to estimate direct connections more accurately than the full covariance matrix. Pcov is the inverse of the covariance matrix:

$$\text{Pcov}\propto {\text{Cov}}^{-1}$$ (7)

#### Regularized partial covariance.

Regularization methods can be implemented during the regression step of calculating partial covariance matrix when the connectivity matrix is sparse. We calculated L1- and L2-regularized partial covariance matrices through the FSL toolbox (Jenkinson, Beckmann, Behrens, Woolrich, & Smith, 2012) using default values for the penalizing parameter: *λ* = 10 for L1-regularization and *ρ* = 0.1 for L2-regularization. For both methods, we varied these regularization parameters, and there was no significant difference in performance over a wide range of values (Supporting Information Figure S8).

#### Granger causality.

Granger causality (Granger, 1969) is a statistical interpretation of causality based on predictability: A is said to “Granger cause” B if the predictability of B declines when A is removed from the predictors. The test of predictability increase or decline typically uses a multivariate vector autoregressive model. We implemented conditional Granger causality (cGranger) through the MVGC MATLAB toolbox (Barnett & Seth, 2014). In this approach, the fundamental assumption is that the time series data is a realization of a stationary vector autoregressive process. The vector autoregressive model order was chosen based on Akaike information criterion and the coefficients of the full/reduced regression model was computed through ordinary least square solution to the regression. For significance test of Granger causality, the G-causality estimator scaled by sample size has an asymptotic *χ*^2^ distribution under the null hypothesis of zero causality. The reported *p* values were before multiple test correction.

#### Differential covariance based methods.

Differential covariance–based methods are a set of algorithms developed to extract direct connections in a network, especially under the influence of common inputs and latent inputs. The calculation of dCov-based FC was detailed in section of main text on Computational Background. In our previous work (Lin et al., 2017, 2020), the advantages of differential covariance–based methods over covariance-based methods have been fully demonstrated both analytically and numerically. Readers can refer to our previous work for more details.

### Significance Testing of FC

To assess the statistical significance of the relationship between hemodynamic signals, we used an autoregressive (AR) bootstrap procedure (Efron & Tibshirani, 1986; Nalci et al., 2019) to preserve the power spectrum density of hemodynamic signals. For a specific connection, denoted as element (*i*, *j*) of the above FC matrices, our null hypothesis was that BOLD signal *z*_*i*_ and *z*_*j*_ were generated independently regardless of other nodes’ time traces. To generate null time series, we fit separate AR processes of model order *q* to node-specific time traces. The model order *q* was determined according to the Bayesian information criterion. A higher order model was rejected if it could not decrease Bayesian information criterion by more than 2. Using the estimated AR coefficients of empirical time series, we generated 1,000 surrogate null time series and then computed the associated functional connectivity corresponding to the null hypothesis. For each connection, we assumed a Gaussian distribution of the null connectivity values. *P* value was calculated as the probability of the empirical FC value that appeared under the null Gaussian distribution. In this paper, we didn’t correct for multiple comparison and instead adopted different significance levels as binarization thresholds.

### Structural Connectivity Matrix

SC matrix was constructed as an *N* × *N* matrix with each node corresponding to the anatomical regions represented by one IC map. To construct the structural connectivity matrix for mouse subjects, we first manually identified the anatomical regions shown on each IC map and then pulled out their connectivity profiles from the existing SC database. If one IC map corresponds to multiple anatomical locations in the database, following the method in Hagmann et al. (2008), we calculated the average connectivity strength of these locations as the connectivity strength of this IC map. The adopted SC database quantified axonal connections within rat cerebral cortex and nuclei (Swanson et al., 2018). Connection strength was quantified from 1 = lowest to 7 = highest. The database was built upon a systematic review of neuro-anatomical literature reporting tracing results. The diagonal of mouse SC matrix was set to 7 because intraregional connections are intensive and there is limited literature reporting intraregional tracing.

For the SC of human subjects, we used the individual level SC matrices constructed in our previous work (Chen et al., 2021; refer to method 4.3 in the reference). In short, we used probabilistic diffusion tractography to evaluate the dMRI measurements (Rosen & Halgren, 2021). The mapping between ICs and dMRI measurements was performed at voxel level. Only 46 HCP components were used in the FC-SC analysis since dMRI measurements were restricted to cortical voxels. Readers could refer to Supporting Information Table S3 (mouse) and Table S2 (HCP) for the identified anatomical regions of each IC map.

### FC and SC Comparison

The comparison of FC and SC was quantified through structual connectivity strength (SCS) and its average (ASCS). SCS refers to the set of structural connectivity strength of the significant functional connections. In practice, we binarized difference FC matrices through their significance level, creating a boolean mask of the SC matrix. The the selected SC values were integrated into the SCS set until such procedure was repeated in all involved subjects. The distribution of the SCS set was plotted in Figure 5A and C. The average value of this SCS set (ASCS) was used as a measure of similarity of SC and FC. ASCS of different methods were plotted in Figure 5B and D.

### Network Topological Analysis

For the HCP dataset, the estimated FC matrices were binarized (threshold = 0.05) and then assessed using topological network measurements so that we could characterize the FC network by using several neuro-biologically meaningful and easily computable measurements (Rubinov & Sporns, 2010). These measurements include node degree (*k*_*i*_), global efficiency (*E*), clustering coefficient (*C*), transitivity (*T*), local efficiency (*E*_*loc*_), and modularity (*Q*). All the measurements were computed using brain connectome toolbox (https://sites.google.com/site/bctnet/). For a binarized network with *N* nodes, these quantities were defined in Table 1 for undirected graphs (Pcov-FC) and directed graphs (Δ*s*-FC): where *a*_*ij*_ ∈ {0, 1} refers to the existence of network edges, *d*_*ij*_ refers to shortest path length between two nodes (*d*_*ij*_ = ∞ if not connected), and *t*_*i*_ is the number of local triangles around node *i*. In the calculation of modularity (*Q*), the network was fully subdivided into a set of nonoverlapping modules *M*, and *e*_*uv*_ is the proportion of all links that connect nodes in module *u* with nodes in module *v*. It can be verified that these measures for undirected graphs are special cases of those for directed graphs in which *a*_*ij*_ = *a*_*ji*_.

**Table 1.** Mathematical definition of network topological measure for (un)directed graphs

Measure	Definition for Undirected Graph	Definition for Directed Graph
(Out)-degree (*k*_*i*_)	∑_*j*∈*N*_ *a*_*ij*_	∑_*j*∈*N*_ *a*_*ij*_
In-degree ($k_{i}^{\text{in}}$)	/	∑_*j*∈*N*_ *a*_*ji*_
Shortest path length (*d*_*ij*_)	∑ *a*_*uv*_, *a*_*uv*_ ∈ *g*_*i*↔*j*_	∑ *a*_*uv*_, *a*_*uv*_ ∈ *g*_*i*→*j*_
Number of triangles (*t*_*i*_)	$\frac{1}{2}$ ∑_*j*,*h*_ *a*_*ij*_*a*_*ih*_*a*_*jh*_	$\frac{1}{2}$ ∑_*j*,*h*_ (*a*_*ij*_ + *a*_*ji*_)(*a*_*ih*_ + *a*_*hi*_)(*a*_*jh*_ + *a*_*hj*_)
Global efficiency (*E*)	$\frac{1}{N}$ ∑_*i*∈*N*_ *E*_*i*_ = $\frac{1}{N}$ ∑_*i*∈*N*_ $\frac{\sum_{j\in N,j\neq i}d_{ij}^{-1}}{N-1}$	
Local efficiency (*E*_*loc*_)	$\frac{1}{N}$ ∑_*i*∈*N*_ $\frac{\sum_{i,j\in N,j\neq h}a_{ij}a_{ih}d_{jh}^{-1}\left(N_{i}\right)}{k_{i}\left(k_{i}-1\right)}$	$\frac{1}{2N}$ ∑_*i*∈*N*_ $\frac{\sum_{i,j\in N,j\neq h}\left(a_{ij}+a_{ji}\right)\left(a_{ih}+a_{hi}\right)\left(d_{jh}^{-1}\left(N_{i}\right)+d_{hj}^{-1}\left(N_{i}\right)\right)}{\left(k_{i}^{\text{out}}+k_{i}^{\text{in}}\right)\left(k_{i}^{\text{out}}+k_{i}^{\text{in}}-1\right)-2\sum_{j}a_{ij}a_{ji}}$
Clustering coefficient (*C*)	$\frac{1}{N}$ ∑_*i*∈*N*_ $\frac{2t_{i}}{k_{i}\left(k_{i}-1\right)}$	$\frac{1}{N}$ ∑_*i*∈*N*_ $\frac{t_{i}}{\left(k_{i}^{\text{out}}+k_{i}^{\text{in}}\right)\left(k_{i}^{\text{out}}+k_{i}^{\text{in}}-1\right)-2\sum_{j}a_{ij}a_{ji}}$
Transitivity (*T*)	$\frac{\sum_{i\in N}2t_{i}}{\sum_{i\in N}k_{i}\left(k_{i}-1\right)}$	$\frac{\sum_{i\in N}t_{i}}{\left(k_{i}^{\text{out}}+k_{i}^{\text{in}}\right)\left(k_{i}^{\text{out}}+k_{i}^{\text{in}}-1\right)-2\sum_{j}a_{ij}a_{ji}}$
Modularity (*Q*)	∑_*u*∈*M*_ [*e*_*uu*_ − (∑_*v*∈*M*_ *e*_*uv*_)]^2^	

To compare the topological properties of different FC matrices, we calculated the correlation *E*, *C*, *T*, *E*_*loc*_, and *Q* between Pcov-FC and Δ*s*-FC across all subjects (Supporting Information Figure S5C). For each individual subject, the node (out)-degree distribution of the Pcov-FC, Δ*s*-FC and and diffusion tensor imaging (Hagmann et al., 2008) networks were fitted by a Gaussian distribution, a power law distribution (small value truncated), and an exponential distribution using maximum likelihood estimation (Supporting Information Figure S5A). For Δ*s*-FC (directed graph), the distributions for the node in-degree and out-degree were very similar and Gaussian distributed (Supporting Information Figure S6). The distributions of $k_{i}^{\text{in}}$ + *k*_*i*_ for directed graphs and 2*k*_*i*_ for undirected graphs are shown in Supporting Information Figure S5A.

To evaluate the relationship between global network efficiency (*E*) and behavioral measurements (BM), we first combined all the BMs documented in the public dataset and restricted dataset (both available by download from the HCP website), resulting in a matrix of size 1003 × 408 = 409,224 (number of subjects *×* number of BMs). Then in reference to Smith et al. (2015), we identified five confounding BMs, including age, weight, height, blood pressure-systolic, blood pressure-diastolic, and total brain volume. All these confounding BMs were regressed out of the network efficiency measurements *E* so that we only focused on the residual efficiency values. We further excluded BMs with more than 200 missing data points. The Pearson correlation between residual efficiency and BMs are calculated, and BMs are sorted according to the significance of their correlation between Δ*s*-FC (Figure 6C).

## SUPPORTING INFORMATION

Supporting information for this article is available at https://doi.org/10.1162/netn_a_00239.

## ACKNOWLEDGMENTS

We thank Dr. Thomas Liu for helpful discussions and for suggesting to look for behavioral correlates of dCov. The authors would like to thank Burke Rosen, Javier How, Robert Kim, Ben Tsuda, and other CNL members for helpful discussion and feedback. The authors also thank Jorge Aldana for assistance with computing resources.

## Note

^1^ Note that the acronym dCov-FC refers to functional connectivity revealed by differential covariance. This should not be confused with the distance covariance based upon pairwise distances between random variables (https://en.wikipedia.org/wiki/Distance_correlation).

## TECHNICAL TERMS

Functional connectivity (FC): Statistical relationship between recorded neural entities; could be inferred using different methods.
Differential covariance (dCov): The novel method of estimating directed functional connectivity, including three matrices described in “Theoretical Background.”
Structural connectivity (SC): Structural connection strength that can be experimentally measured. Specifically, it refers to tracing strength in the mouse dataset and fiber density in the HCP dataset.
Node: Neural entity being recorded. Here, it refers to a group of voxels belong to the same independent component.
Average structural connectivity strength (ASCS): Average of SCS. This was used as a measure of similarity between FC and SC.
Structural connectivity strength (SCS): The set of structural connectivity strength of the statistically significant functional connections.
Network efficiency: Average of the inverse path length of the shortest path between two nodes. A measure of network integration.
