# Supplementary material for: Functional connectivity of fMRI using differential covariance predicts structural connectivity and behavioral reaction times
Source: Netw Neurosci. 2022 Jun 1;6(2):614–33. doi: 10.1162/netn_a_00239 (PMC9207998; doi:10.1162/netn_a_00239)
Supplement: Supplementary file 1 [file netn-06-614-s001.pdf]

Supplementary: Functional connectivity of fMRI using differential covariance predicts structural connectivity and behavioral reaction times

Yusi Chen, Qasim Bukhari, Tiger W. Lin, Terrence Sejnowski

Variable	Annotation	HCP (B=3T)	Mouse (B=9.4T)
ϑ_0	Frequency offset at the outer surface of the magnetized vessel for fully deoxygenated blood	80.6 [2]	252.5 [2]
ρ	Oxygen extraction fraction	0.8	0.8
ϵ	Ratio of intra- and extravascular signal	0.5	0.5
r_0	Slope of the relation between intravascular relaxation rate and oxygen saturation	167 [4]	458 [1]
TE	Time of echo (ms)	30	40
τ	haemodynamic transit time	0.98	0.98
α	Grubbs exponent	0.32	0.32
κ	Rate of signal decay	0.65	0.65
γ	Rate of flow-dependent elimination	0.41	0.41
η	Neuronal efficacy	0.54	0.54
V_0	Resting blood volumne fraction	0.02	0.02

Table S1: Parameters used in backward reconstruction

Table S2: **Annotated components for HCP dataset.** First column is the index number of pre-selected IC components. Second column is the manually registered anatomical region. Third column is the assigned sub-network. DMN: default mode network

Component number	Anatomical Region	Sub-network
1	Occipital Pole	Visual Network
2	Inferior Parietal Lobe	DMN
3	Lateral Occipital Cortex	Visual Network
4	Cuneal Cortex/Occipital Pole	Visual Network
5	Supramarginal Gyrus	DMN
6	Lateral Occipital Cortex	Visual Network
7	Supramarginal Gyrus	DMN
8	Lateral Occipital Cortex	Visual Network
9	Inferior Parietal Lobe	DMN
10	Medial Prefrontal Cortex/Anterior Cingulate Cortex/Lateral Temporal Cortex	DMN
11	Lingual Gyrus/medial occipitotemporal gyrus	Visual Network
12	Angular Gyrus	DMN
13	Occipital Pole	Visual Network
14	Lateral Occipital Cortex - Left	Visual Network
15	Precuneous Cortex	Other Network
16	Occipital Pole	Visual Network
17	Lingual Gyrus/medial occipitotemporal gyrus	Visual Network
18	Lateral Occipital Cortex	Visual Network
19	Occipital Pole	Visual Network
20	Inferior Parietal Lobe	DMN
21	Precentral Gyrus	sensorimotor network
22	Orbital Frontal Cortex	DMN
23	Postcentral Gyrus	sensorimotor network
24	Lateral Occipital Cortex - Right	Visual Network
25	Occipital Pole - Left	Visual Network
26	Frontal Pole	Attention Network
27	Superior Parietal Lobule	Attention Network
28	Hippocampus/Parahippocampal Cortex	DMN
29	Lateral Temporal Cortex	DMN
30	Lateral Occipital Cortex	Visual Network
31	Orbital Frontal Cortex/Lateral Temporal Cortex	Other Network
32	Occipital Pole	Visual Network
33	Inferior Parietal Lobe – Left	DMN
34	Lateral Occipital Cortex	Visual Network
35	Postcentral Gyrus	sensorimotor network
36	Medial Prefrontal Cortex/Anterior Cingulate Cortex	DMN
37	Orbital Frontal Cortex/Lateral Temporal Cortex	DMN
38	Precentral Gyrus / Juxtapositional Lobule Cortex (formerly Supplementary Motor Cortex)	sensorimotor network
39	Occipital Pole	Visual Network
40	Middle Frontal Gyrus	Attention Network
41	Postcentral Gyrus - Left	sensorimotor network
43	Postcentral Gyrus - Right	sensorimotor network
44	Orbital Frontal Cortex	DMN
45	Superior Temporal Gyrus	sensorimotor network
48	Frontal Pole	Attention Network
49	Lateral Occipital Cortex	Visual Network
50	Occipital Fusiform Gyrus	Visual Network
60	Hippocampus/Parahippocampal Cortex	DMN
64	Hippocampus/Parahippocampal Cortex	DMN
67	Thalamus	Thalamic Network
69	Putamen	sensorimotor network
76	Putamen - Right	sensorimotor network
78	Right V1	Visual Network
82	Left V1	Visual Network
84	Visual Cortex	Visual Network
87	Thalamus	Thalamic Network
92	Right V1	Visual Network
93	Left V1	Visual Network
96	Thalamus - Right	Thalamic Network
97	Thalamus - Left	Thalamic Network

Table S3: **Annotated IC maps for the Bukhari mouse dataset.** First column is the index of pre-selected IC components. Second column is the manually registered anatomical region. Third column is the corresponding anatomical locations in the Swanson database [3]. SSp = Primary somatosensory area , SSs = Supplemental somatosensory area, MO = Somatomotor areas, ACAd(v) = Anterior cingulate area dorsal (ventral), RSPd(v) = Retrosplenial region dorsal (ventral), CEA = Central amygdalar nucleus, MEA = Medial amygdalar nucleus, AAA = Anterior amygdalar area, BMA = Basomedial amygdalar nucleus, PAA = Piriform-amygdalar area, IA = Intercalated amygdalar nuclei, AAA = Anterior amygdalar area, BLA = Basolateral amygdalar nucleus, LA = Lateral amygdalar nucleus, PA = Posterior amygdalar nucleus, ECT = Ectorhinal area, PERI = Perirhinal area, CP = Caudoputamen, AI = Agranular insular, PIR = Piriform area, GP = Globus pallidus, NA: no corresponding anatomical locations in the database

IC number	Full Name	Swanson region (Abbreviations)	Subnetwork
1	Primary Somatosensory Cortex (both side)	SSp	Lateral cortical network
2	Secondary Sematosensory Cortex (right)	SSs	Lateral cortical network
3	Secondary Sematosensory Cortex (left)	SSs	Lateral cortical network
4	Primary Somatosensory Cortex (right)	SSp	Lateral cortical network
5	Primary Somatosensory Cortex (left)	SSp	Lateral cortical network
6	Motor Cortex (right)	MO	Lateral cortical network
7	Motor Cortex (left)	MO	Lateral cortical network
8	Anterior Cingulate Cortex, dorsal	ACAd	Default mode network
9	Cingulate Cortex/ Retrosplenial Cortex	ACAv/ACAd/RSPd/RSPv	Default mode network
10	Amygdalar (right)	CEA/MEA/AAA/BMA/PAA/IA/AAA/BLA/LA/PA	Associative cortical network
11	Amygdalar (left)	CEA/MEA/AAA/BMA/PAA/IA/AAA/BLA/LA/PA	Associative cortical network
12	Ectorhinal Cortex (left)	ECT	Associative cortical network
13	Ectorhinal Cortex (right)	ECT	Associative cortical network
14	Perirhinal Area (right)	PERI	Associative cortical network
15	Caudoputamen (right)	CP	Subcortical network
16	Caudoputamen (left)	CP	Subcortical network
17	Insular (left)	AI	Associative cortical network
18	Insular (right)	AI	Associative cortical network
19	Insular (left)	AI	Associative cortical network
20	Insular (right)	AI	Associative cortical network
21	Perirhinal Area (right)	PERI	Associative cortical network
22	Perirhinal Area (left)	PERI	Associative cortical network
23	Piriform Cortex (left)	PIR	Associative cortical network
24	Piriform Cortex (right)	PIR	Associative cortical network
25	Globus Pallidus (right)	GP	Subcortical network
26	Globus Pallidus (left)	GP	Subcortical network
27	Thalamus, dorsal (left)	NA	Thalamic network
28	Thalamus, dorsal (right)	NA	Thalamic network
29	Thalamus, ventral (left)	NA	Thalamic network
30	Thalamus, ventral (right)	NA	Thalamic network

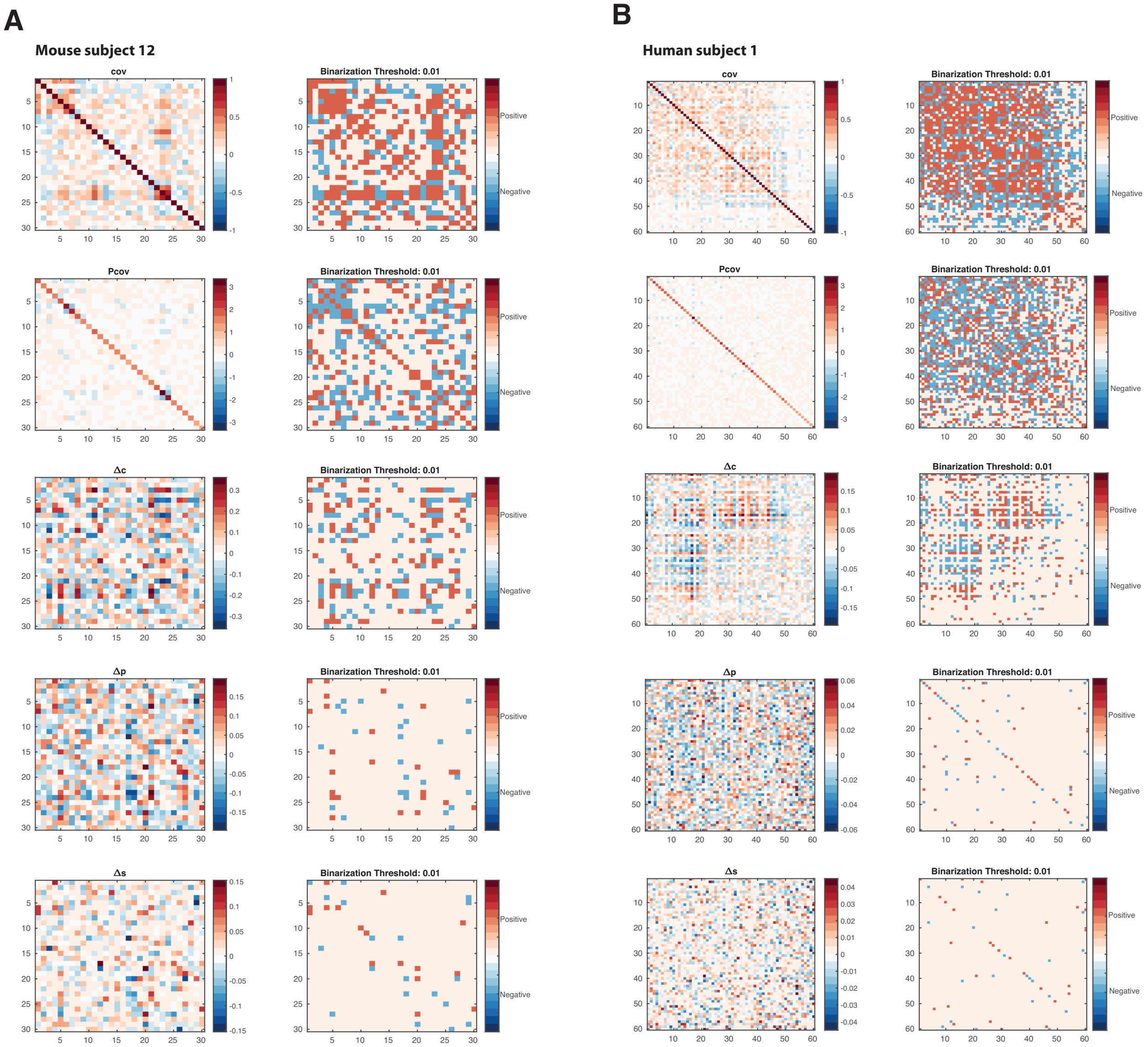

Figure S1: **Differential covariance based methods produce sparse and directed FC.** Example covariance matrix (cov), precision matrix (Pcov), Δc, Δp and Δs and their binarized matrices (right) of one mouse subject (A) and one human subject (B).

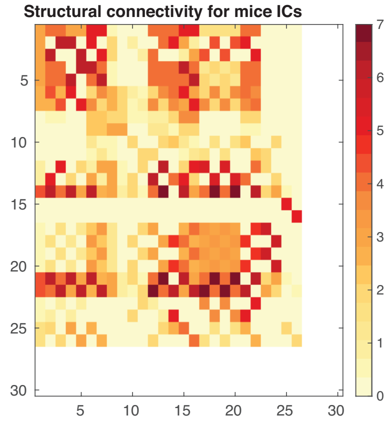

Figure S2: **The structural connectivity matrix for mouse**

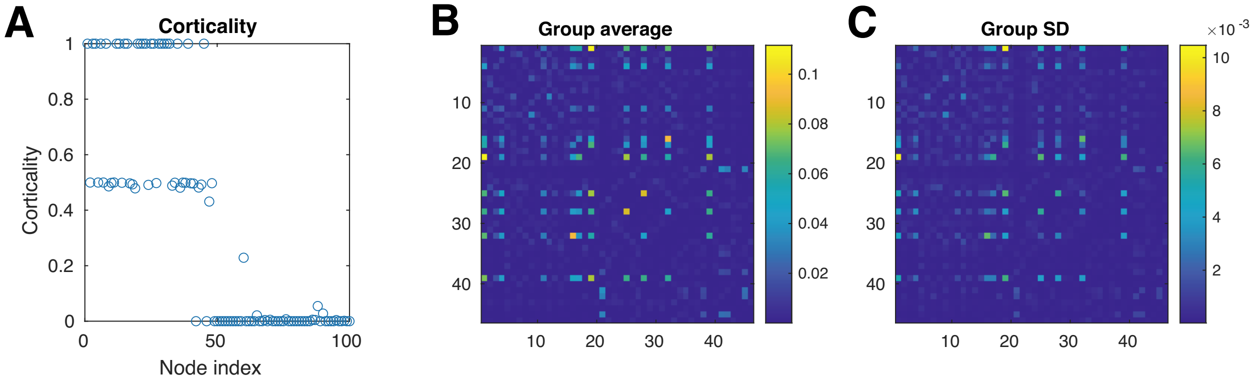

Figure S3: **Individual level dMRI statistics.** (A) Corticality was defined as the proportion of cortical voxels within each IC. Since dMRI measurements are only available for cortical surface voxels. Our analysis was restricted to the first 46 ICs with corticality greater than 40%. (B) Average of the dMRI matrices across the entire 998 subjects. (C) Standard deviation.

A Mouse subject 2, time traces of IC20 and IC27

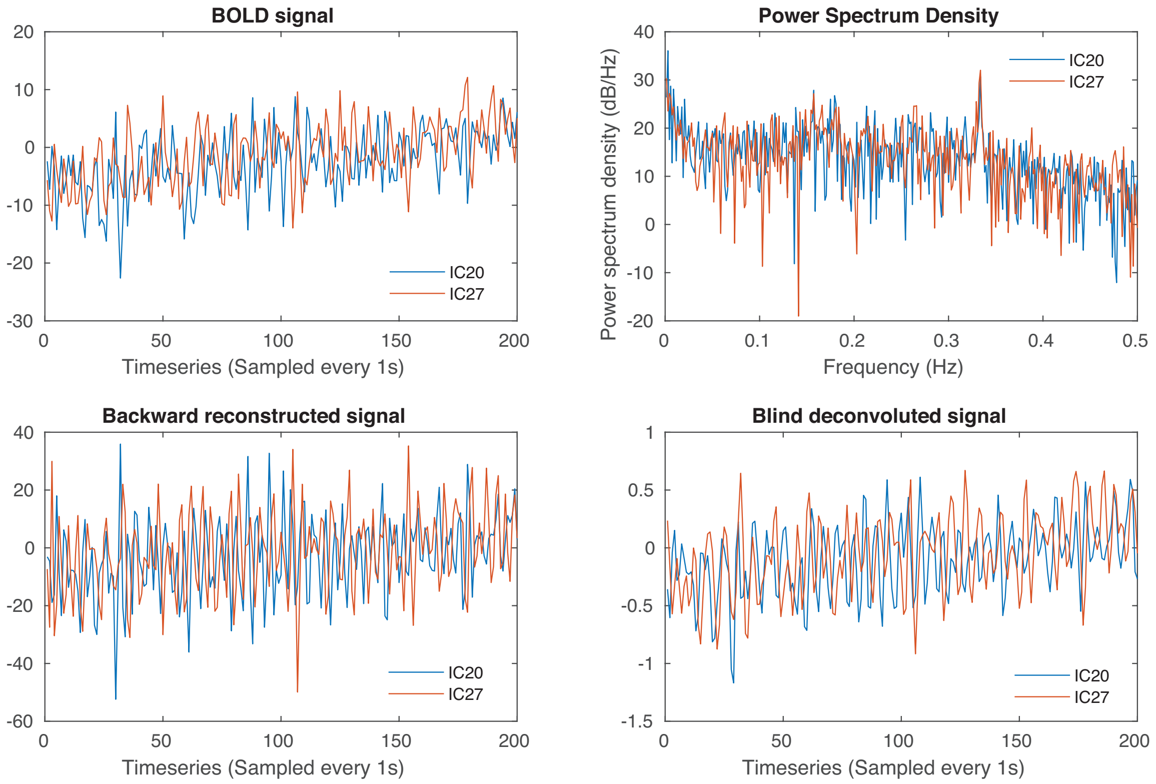

B Human subject, time traces of IC45 and IC48

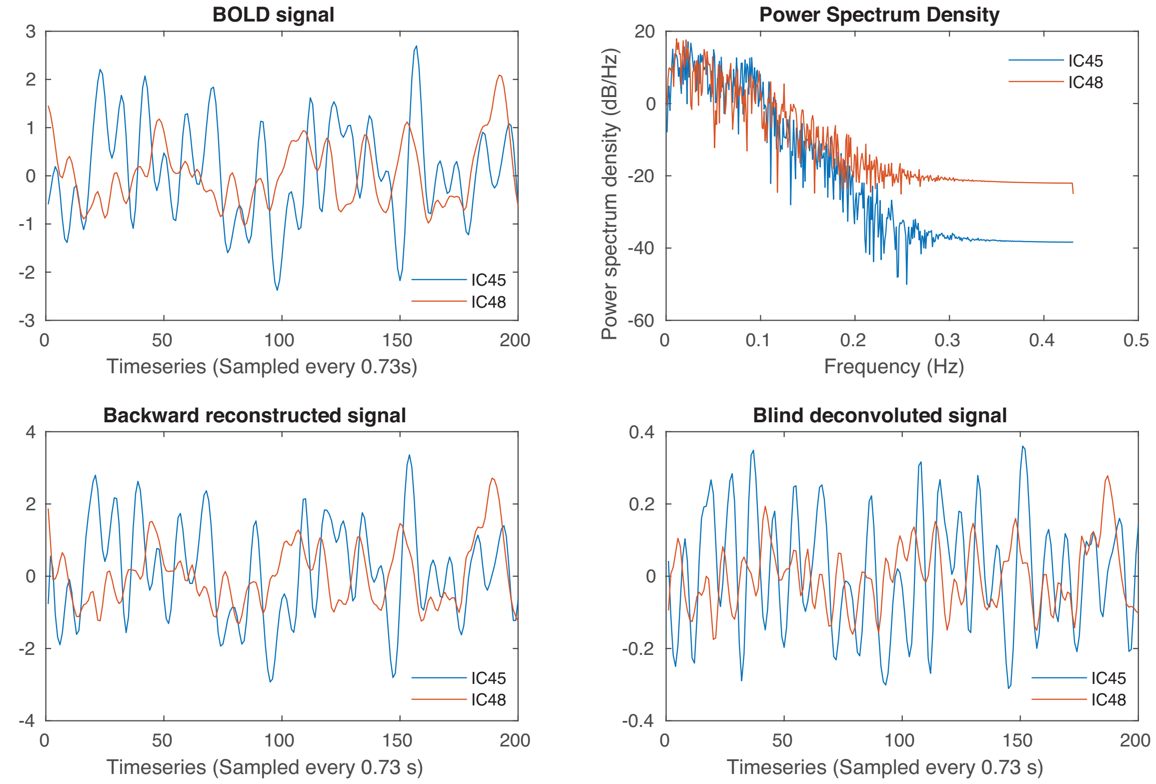

Figure S4: **Two example time traces from one mouse subject (A) and one human subject (B)** Upper left: haemodynamic signal after dual regression; Upper right: power spectrum density of the haemodynamic signal; Lower left: backward reconstructed signal; Lower right: blind deconvoluted signal

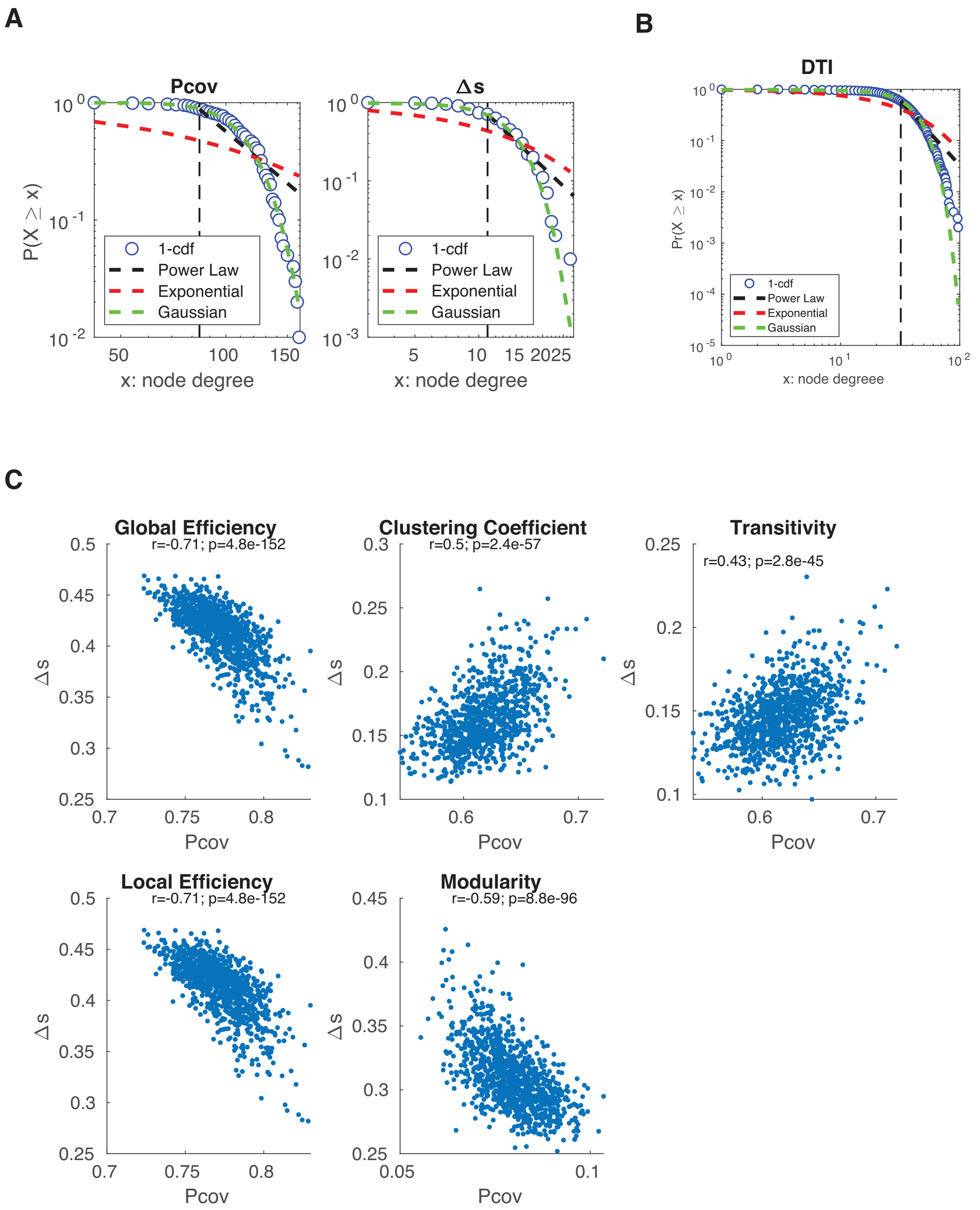

Figure S5: **Network topology analysis of FC.** (A) Cumulative density function of network degree distribution of Pcov-FC and Δs-FC from one HCP subject. (B) Network degree distribution of structural connectivity matrix from diffusion tensor imaging (DTI). (C) Scatterplots of global efficiency, local efficiency, clustering coefficient, transitivity and modularity of Δs-FC versus that of Pcov-FC.

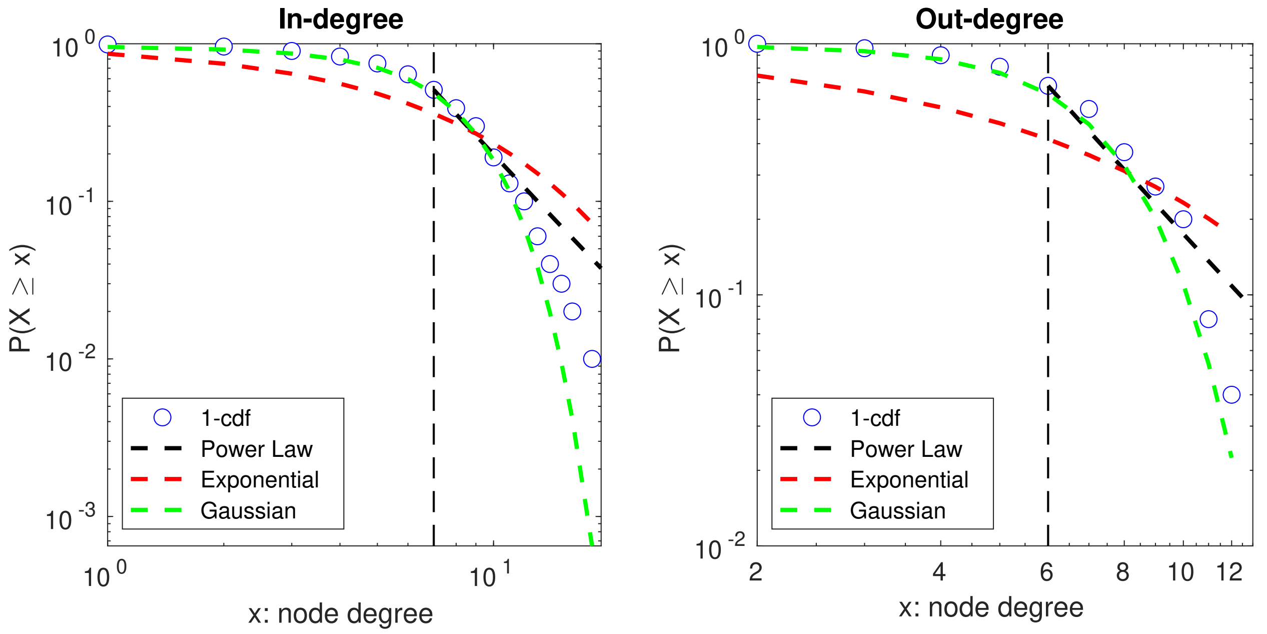

Figure S6: **In-/Out- degree node distribution for Δs-FC**

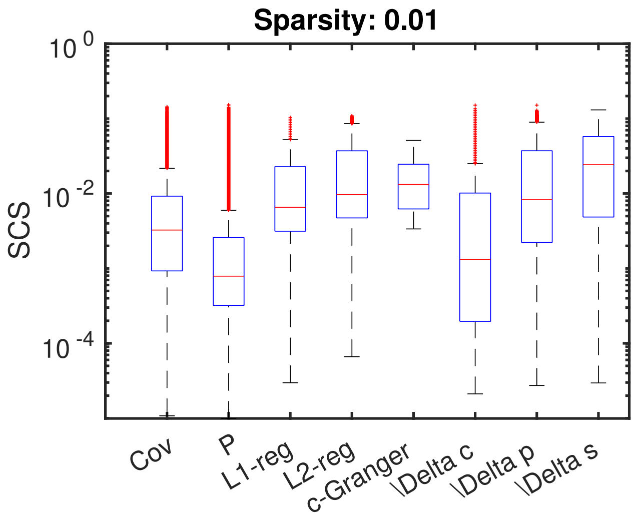

Figure S7: **SCS of all subjects inferred from different methods thresholded to a same sparsity level.** Δs still have significantly higher (ranksum test, $p < 10^{-34}$) SCS values compared to all other methods. \Delta stands for Δ

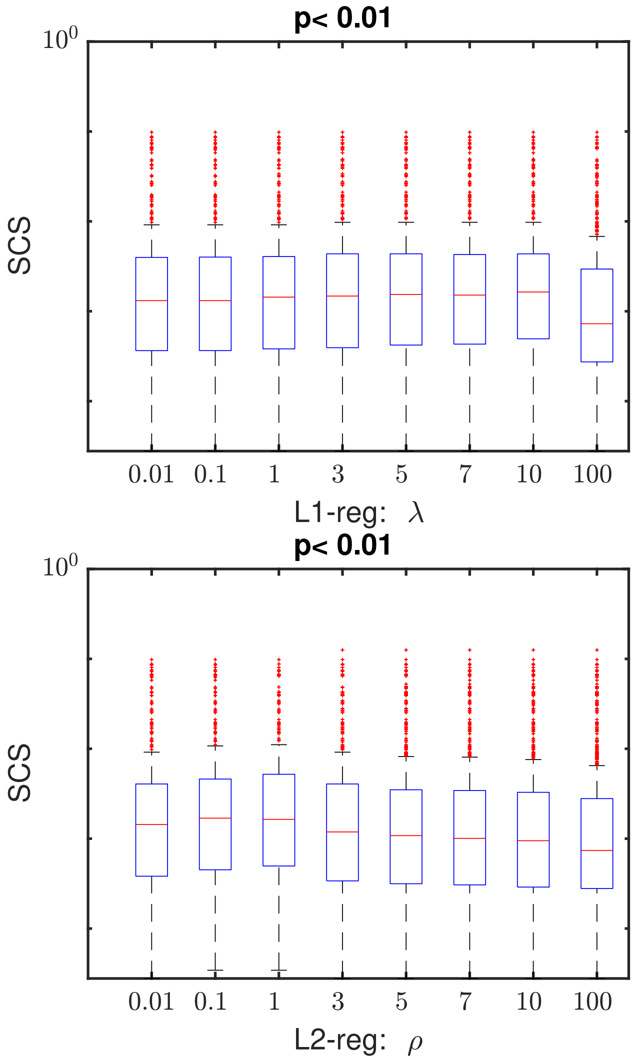

Figure S8: SCS of one HCP subject inferred by L1-/L2-reg penalized to different extents (λ and ρ). The default value used in the main text is $\lambda = 10$ and $\rho = 0.1$. All other parameters showed similar (no statistical difference) or worse performance. The binarization threshold was $p < 0.01$.

References

[1] Sang-Pil Lee, Afonso C Silva, Kamil Ugurbil, and Seong-Gi Kim. Diffusion-weighted spin-echo fmri at 9.4 t: microvascular/tissue contribution to bold signal changes. *Magnetic Resonance in Medicine: An Official Journal of the International Society for Magnetic Resonance in Medicine*, 42(5):919–928, 1999.

[2] S Ogawa, RS Menon, David W Tank, SG Kim, H Merkle, JM Ellermann, and K Ugurbil. Functional brain mapping by blood oxygenation level-dependent contrast magnetic resonance imaging. a comparison of signal characteristics with a biophysical model. *Biophysical journal*, 64(3):803–812, 1993.

[3] Larry W Swanson, Joel D Hahn, Lucas GS Jeub, Santo Fortunato, and Olaf Sporns. Subsystem organization of axonal connections within and between the right and left cerebral cortex and cerebral nuclei (endbrain). *Proceedings of the National Academy of Sciences*, 115(29):E6910–E6919, 2018.

[4] Jason M Zhao, Chekesha S Clingman, M Johanna Närväinen, Risto A Kauppinen, and Peter CM van Zijl. Oxygenation and hematocrit dependence of transverse relaxation rates of blood at 3t. *Magnetic Resonance in Medicine: An Official Journal of the International Society for Magnetic Resonance in Medicine*, 58(3):592–597, 2007.
